# THE EMOTION PARADOX IN THE AGING BODY AND BRAIN

**DOI:** 10.1093/geroni/igad104.0220

**Published:** 2023-12-21

**Authors:** Mara Mather

**Affiliations:** University of Southern California, Los Angeles, California, United States

## Abstract

With age, parasympathetic activity decreases while sympathetic activity increases. Thus, the typical older adult has low heart rate variability (HRV) and high noradrenergic activity. Younger adults with this physiological profile tend to be chronically unhappy and stressed. Yet, with age, emotional experience tends to improve. Why doesn’t older adults’ emotional well-being suffer as their HRV decreases? To address this apparent paradox, I present the autonomic compensation model. In this model, failing organs and initial phases of Alzheimer’s pathology trigger noradrenergic hyperactivity in older adults. Older brains attempt to compensate by increasing autonomic regulatory processes in ventromedial prefrontal (vmPFC) cortex. Age-related declines in nerve conduction reduce the ability of these compensatory attempts to reduce hyperactive noradrenergic activity and increase peripheral HRV. However, despite their muted effects on peripheral autonomic activity, these autonomic compensation attempts bias brain processing in favor of stimuli that tend to increase parasympathetic activity and against stimuli that tend to increase sympathetic activity. The autonomic compensation model can account for the “age-related positivity effect” in which, compared with younger adults, older adults show attentional and memory biases favoring positive over negative stimuli. Consistent with the autonomic compensation model, the age-related positivity effect is associated with vmPFC activity and functional connectivity and is evident even in early attention processes resistant to goal-directed processing. The autonomic compensation model posits that the aging brain responds to sympathetic hyperactivity in ways that amplify on-going automatic emotion regulation processes and so help maintain emotional well-being.

